# Validation of a Quantitative Cervical Vertebral Bone Age Model in North Indian Children and Adolescents: A Cross-Sectional Study

**DOI:** 10.7759/cureus.98902

**Published:** 2025-12-10

**Authors:** Chand Sawhney, Shubham Gupta, Gaurav Dhir, Mohammad Haroon Ahmed, Neetu Dabla, Saurabh S Parihar, Seema Gupta

**Affiliations:** 1 Department of Orthodontics, Maharana Pratap College of Dentistry and Research Centre, Gwalior, IND; 2 Department of Orthodontics, Kothiwal Dental College and Research Centre, Moradabad, IND

**Keywords:** age, cervical vertebrae, children, quantitative, skeletal

## Abstract

Introduction: The assessment of skeletal maturity is essential for orthodontic treatment planning and evaluation in growing patients. The present study aimed to validate the accuracy and reliability of quantitative cervical vertebral bone age (CVBA) regression formulae in a North Indian population, using the Fishman hand-wrist method as the reference standard.

Materials and methods: This retrospective cross-sectional study included 180 North Indian children (90 males and 90 females) aged 4-18 years with pre-treatment digital lateral cephalometric and left-hand-wrist radiographs taken on the same day. Eleven geometric measurements of the C2-C4 vertebrae were performed using the Dolphin Imaging software. Previously published sex-specific regression equations were applied to derive continuous CVBA values. Hand-wrist skeletal age was determined and coded from hand-wrist radiographs independently by two blinded, calibrated orthodontists using the Fishman method and converted to decimal years. Chronological age was calculated from birth records. The reliability of the measurements, correlation, agreement (Bland-Altman), and differences among the three age estimates were statistically analyzed.

Results: Mean chronological age was 11.95 ± 1.78 years, formula-derived CVBA 12.03 ± 1.74 years, and Fishman hand-wrist skeletal age 12.02 ± 1.77 years (repeated-measures ANOVA, p = 0.882). Pearson correlation coefficients exceeded 0.915 (mostly > 0.988) for all pairwise comparisons. Bland-Altman analysis revealed negligible bias (+0.01 years) between CVBA and Fishman skeletal age, with 95% limits of agreement of −0.48 to +0.50 years. Intra- and inter-examiner ICCs for vertebral measurements were >0.94 and >0.92, respectively, for CVBA and hand-wrist skeletal age. No significant differences were found across sex or age subgroups.

Conclusions: The quantitative CVBA model exhibited outstanding accuracy, reliability, and interchangeability with the Fishman hand-wrist method in North Indian children and adolescents. Utilizing routinely acquired lateral cephalograms provides a reproducible, objective, and radiation-efficient alternative for assessing skeletal maturity, thereby justifying their routine clinical implementation in this population.

## Introduction

Assessment of skeletal maturity is fundamental in pediatric endocrinology, orthodontics, and forensic medicine for diagnosing growth disorders, timing orthodontic interventions, and estimating chronological age in individuals without reliable documentation [1]. Among the various skeletal indicators, cervical vertebral maturation (CVM) has gained prominence since its introduction. The CVM stages have been modified and improved several times. The 2005 classification system developed by Franchi and McNamara [2] demonstrated high compliance with the hand-wrist method. Unlike hand-wrist radiographs, which require additional exposure to ionizing radiation, cervical vertebrae are routinely visible on lateral cephalograms taken for orthodontic diagnosis, making CVM methods radiation-efficient and clinically convenient [3].

Traditional CVM approaches rely on qualitative morphological descriptors, such as the concavity of the lower border and vertebral body shape of the C2, C3, and C4 cervical vertebrae [3]. Despite widespread adoption, these methods suffer from significant inter- and intra-observer variability, moderate to low reproducibility, and subjectivity in assigning stages, particularly in transitional phases [4]. To overcome these limitations, quantitative approaches using linear, angular, and area measurements or regression equations have been proposed [5,6]. However, many existing quantitative models depend on manual landmark identification (prone to measurement error), use limited sample sizes, lack robust statistical validation on independent datasets, or do not provide a continuous bone age equivalent that can be directly compared with established standards, such as Greulich-Pyle or Tanner-Whitehouse.

The advent of advanced artificial intelligence models has enabled the development of fully automated or semi-automated, highly reproducible, and objectively validated quantitative systems [7]. Although AI-based models can achieve high accuracy on training data, they often operate as "black boxes" with limited interpretability and the risk of overfitting or bias when applied to diverse populations, necessitating transparent, advanced statistical methods (such as regression-based or hybrid models) to ensure clinical trustworthiness and generalizability. Therefore, a new quantitative cervical vertebral bone age (CVBA) model that integrates multiple geometric indices and accounts for sexual dimorphism was proposed by Chandrasekar et al. [8] and validated against both chronological age and peak height velocity.

This study aimed to assess the accuracy and reliability of quantitative CVBA prediction formulae when applied to a North Indian population using the Fishman hand-wrist skeletal maturity assessment method as the reference standard. The primary objective of this study was to evaluate the agreement and accuracy of the quantitative formula-derived CVBA with the Fishman hand-wrist skeletal age method in North Indian children and adolescents. The secondary objectives were to assess the measurement reliability and to examine the performance of the CVBA method across sex- and age-based subgroups.

## Materials and methods

Study design and setting

This retrospective cross-sectional diagnostic accuracy study was conducted at the Department of Orthodontics, Maharana Pratap College of Dentistry and Research Centre, Gwalior, India. Digital archival records from January 2015 to December 2023 were obtained. The study protocol was approved by the Institutional Ethics Committee (approval number: MPCD&RC/IEC/2024/03). As this study involved fully anonymized retrospective radiographs with no additional radiation exposure or patient interactions, the requirement for individual informed consent was waived.

Eligibility criteria

Inclusion criteria included individuals of North Indian ancestry aged 4.0-18.0 years with high-quality pre-treatment digital lateral cephalometric and left-hand-wrist radiographs taken on the same day (±7 days), showing clear visualization of C2-C4 vertebrae and all hand-wrist ossification centers. Exclusion criteria included previous orthodontic treatment, craniofacial syndromes, cervical vertebral anomalies/fusions, systemic endocrine or growth disorders, hormonal therapy, trauma or surgery to the neck/hand/wrist, image distortion, or head rotation/tilt exceeding 5°.

Sample size and allocation

The sample size was estimated using G*Power software version 3.1.9.2 (Heinrich Heine University, Düsseldorf, Germany). Assuming a minimum anticipated correlation coefficient of 0.70 between CVBA and skeletal age [8], with 80% statistical power and a two-sided alpha of 0.05, the required minimum sample size was 172. To compensate for potential exclusions and to improve subgroup analyses, a total sample of 180 subjects was finally included.

Acquisition of radiographs

All lateral cephalometric and hand-wrist radiographs were obtained using a standardized digital radiography unit (Planmeca ProMax®, Planmeca Oy, Helsinki, Finland). Lateral cephalograms were acquired with the patient in natural head position, teeth in centric occlusion, and lips relaxed. The exposure parameters were set at 68-72 kVp, 8-10 mA, and 12-16 seconds, depending on patient size. Hand-wrist radiographs were acquired using the same unit with a standardized focus-film distance of 100 cm and exposure parameters of 50-55 kVp and 4-6 mA. All images were captured using a flat-panel digital detector with a spatial resolution of ≥7 lp/mm and exported in uncompressed TIFF format for analysis. Routine equipment calibration was performed in accordance with the manufacturer’s guidelines.

Formula-derived skeletal age assessment

All lateral cephalometric radiographs were exported as uncompressed TIFF files and analyzed using Dolphin Imaging Premium software version 11.95 (Dolphin Imaging & Management Solutions, Chatsworth, CA, USA) on a 24-inch diagnostic-grade monitor under standardized lighting conditions. A single calibrated investigator placed all landmarks and measured the second (C2), third (C3), and fourth (C4) cervical vertebrae according to the landmark definitions published by Chandrasekar et al. [8]. Radiographic magnification was corrected using an embedded 10-cm reference scale. The recorded variables included anterior vertebral body height (AH2, AH3, and AH4), posterior vertebral body height (PH2, PH3, and PH4), anteroposterior vertebral body length (AP2, AP3, and AP4), and depth of concavity on the inferior border of each vertebra (D2, D3, and D4). All the necessary ratio variables were automatically computed using the software. The published sex-specific regression equations of Chandrasekar et al. [8] were then directly applied to these measurements to calculate a continuous formula-derived CVBA expressed in decimal years for each participant.

Reliability of measurements

Intra-examiner reliability was assessed by the primary investigator, who repeated all measurements on 30 randomly selected radiographs after a four-week interval. Inter-examiner reliability was evaluated by having a second calibrated orthodontist independently perform the exact measurements on these 30 radiographs. Reproducibility was quantified using intraclass correlation coefficients (ICC; two-way random effects model, absolute agreement). The results demonstrated excellent reliability. The intra-examiner ICC values ranged from 0.962 to 0.998 for linear measurements, 0.949 to 0.987 for concavity depths, and 0.941 to 0.978 for the ratio variables. The inter-examiner ICC values ranged from 0.945 to 0.996 for linear measurements, 0.932 to 0.981 for concavity depths, and 0.929 to 0.974 for ratios.

Reference standard: hand-wrist skeletal age assessment

Hand-wrist skeletal age was independently determined by two experienced and blinded orthodontists using the Fishman Skeletal Maturity Assessment method [9]. This method evaluates 11 discrete maturity stages at six anatomical sites: the adductor sesamoid of the thumb, the epiphyses of the proximal phalanges of the third and fifth fingers, the epiphyses of the middle phalanges of the third and fifth fingers, and the distal radius. Disagreements were resolved through consensus. The final Fishman stage was converted to the skeletal age in decimal years using the standard Fishman conversion table [9]. Inter-examiner agreement for stage assignment and converted skeletal age was assessed using weighted kappa and ICC, respectively. The results showed excellent inter-examiner agreement. The weighted kappa for the Fishman stage assignment was 0.94, indicating almost perfect agreement. The ICC for converted skeletal age in decimal years was 0.97.

Chronological age

The chronological age at the time of radiography was calculated in decimal years as the difference between the radiographic date and the date of birth recorded in the participant’s clinical record.

Statistical analysis

Data were analyzed using SPSS Statistics version 26.0 (IBM Corp. Released 2019. IBM SPSS Statistics for Windows, Version 26.0. Armonk, NY: IBM Corp.). Age data were assessed for normality using the Shapiro-Wilk test and were found to be normally distributed. Data are presented as the mean ± standard deviation. The three age assessment methods were compared using repeated-measures analysis of variance (ANOVA). Agreement between methods was evaluated using Bland-Altman analysis, and their relationships were quantified using Pearson's correlation coefficient. For Bland-Altman analysis, a priori clinically acceptable limits of agreement were defined as ±1.0 years, based on accepted orthodontic thresholds for assessing skeletal maturity. A p-value less than 0.05 was considered statistically significant for all tests.

## Results

The study population was divided into three cohorts based on age, with 60 (33.33%) participants in each cohort. The overall population comprised 180 participants: 90 (50%) male and 90 (50%) female (Table 1).

**Table 1:** Distribution of the study sample according to sex and chronological age cohorts Values are presented as frequency (n) and percentage (%).

Variables	Category	Cohort 1 (n = 60, 33.33%) (4-8 years)	Cohort 2 (n = 60, 33.33%) (9- 13 years)	Cohort 3 (n = 60, 33.33%) (14-18 years)
Sex	Male (n = 90)	30 (50%)	30 (50%)	30 (50%)
	Female (n = 90)	30 (50%)	30 (50%)	30 (50%)

A comparison between chronological age, formula-derived CVBA, and hand-wrist skeletal age revealed no statistically significant differences across the study groups. A repeated measures ANOVA for the overall cohort (n = 180) showed that the mean ages across the three methods were nearly identical (chronologic: 11.95 ± 1.78, formula-derived CVBA: 12.03 ± 1.74, hand-wrist skeletal age: 12.02 ± 1.77 years), which was confirmed by a non-significant difference (p = 0.882). Therefore, it can be inferred that the formula-derived CVBA and hand-wrist skeletal age provide age estimates that are not significantly different from each other or from the chronological age in this sample, suggesting high concordance among the three methods for assessing age in this population (Table 2).

**Table 2:** Comparison of chronological age, formula-derived CVBA, and Fishman hand-wrist skeletal age p > 0.05 denotes no statistical insignificance using repeated-measures ANOVA. Values are presented as mean ± SD. ANOVA: analysis of variance, CVBA: cervical vertebral bone age, SD: standard deviation

Sex	n	Chronologic age (years)	Formula-derived CVBA (years)	Hand-wrist skeletal age (years)	F stats	p-value
Male	90	11.93 ± 1.79	12.03 ± 1.75	12.00 ± 1.79	1.24	0.922
Female	90	11.72 ± 2.24	12.03 ± 1.74	12.02 ± 1.75	0.87	0.471
Overall	180	11.95 ± 1.78	12.03 ± 1.74	12.02 ± 1.77	0.36	0.882

The comparison of age assessment methods across different chronological age cohorts revealed no statistically significant differences between chronological, formula-derived CVBA, and hand-wrist skeletal age in any subgroup. As detailed in Table 3, the repeated-measures ANOVA yielded non-significant p-values for all cohorts, both in the overall analysis and when stratified by sex. While minor variations in mean ages were observed, for instance, in the overall cohort 1, the formula-derived CVBA (6.55 ± 1.57 years) was slightly higher than the hand-wrist skeletal age (5.95 ± 2.07 years); these differences did not reach statistical significance. Similarly, in cohort 3 males, the chronological age (15.34 ± 1.89 years) trended higher than the hand-wrist skeletal age (14.84 ± 1.78 years), but again, the difference was not significant. Therefore, within the confines of this study, the formula-derived CVBA and hand-wrist skeletal age assessment method yielded results that were not significantly different from chronological age or from each other across all age groups and sexes (Table 3).

**Table 3:** Comparison of the three age estimation methods across chronological age cohorts and by sex p > 0.05 denotes no statistical insignificance using repeated-measures ANOVA. Values are presented as mean ± SD. ANOVA: analysis of variance, CVBA: cervical vertebral bone age, SD: standard deviation

Variable	Category	Chronologic age (years)	Formula-derived CVBA (years)	Hand-wrist skeletal age (years)	F stats	p-value
		Mean ± SD	Mean± SD	Mean± SD		
Overall	Cohort 1	6.25 ± 1.87	6.55 ± 1.57	5.95 ± 2.07	2.37	0.095
	Cohort 2	11.45 ± 2.16	11.15 ± 1.86	11.78 ± 2.31	2.95	0.059
	Cohort 3	16.18 ± 1.98	15.98 ± 2.08	16.68 ± 2.18	1.89	0.160
Male	Cohort 1	5.24 ± 1.75	5.54 ± 1.15	5.95 ± 2.15	3.01	0.061
	Cohort 2	10.23 ± 1.16	10.53 ± 1.86	10.03 ± 1.66	2.26	0.106
	Cohort 3	15.34 ± 1.89	15.14 ± 1.49	14.84 ± 1.78	1.90	0.150
Females	Cohort 1	6.34 ± 1.25	6.64 ± 1.15	6.56 ± 1.15	0.45	0.642
	Cohort 2	11.88 ± 2.26	12.08 ± 1.76	11.42 ± 2.16	2.40	0.092
	Cohort 3	16.75 ± 2.15	16.35 ± 2.45	16.15 ± 2.32	2.12	0.132

All pairwise correlations yielded coefficients (r) exceeding 0.915, with most exceeding 0.988, indicating a near-perfect linear relationship. This pattern of strong concordance was consistently observed in the overall sample and was maintained when the data were stratified by sex. These results suggest that formula-derived CVBA and hand-wrist skeletal age are remarkably consistent with chronological age and with each other. This high level of agreement suggests that, within the context of this study, these methods can be used interchangeably as highly reliable indicators of age (Table 4).

**Table 4:** Pearson correlation coefficients (r) between chronological age, formula-derived CVBA, and Fishman hand-wrist skeletal age in the overall sample and by sex Values are Pearson’s correlation coefficient (r) with 95% CI; r = 0.90–1.00 indicates a very strong correlation. CVBA: cervical vertebral bone age, CI: confidence interval

Pairwise correlation	Overall		Male		Female	
	r value	CI at 95%	r value	CI at 95%	r value	CI at 95%
Chronologic age vs. formula-derived CVBA age	0.915	0.888-0.990	0.990	0.984-0.994	0.988	0.981-0.992
Chronologic age vs. hand-wrist skeletal age	0.918	0.892-0.938	0.988	0.981-0.992	0.989	0.982-0.993
Formula-derived CVBA vs. hand-wrist skeletal age	0.990	0.987-0.992	0.990	0.984-0.994	0.988	0.981-0.992

The formula-derived CVBA showed excellent agreement with the Fishman hand-wrist skeletal age method, with a clinically negligible bias of only +0.01 years and narrow 95% limits of agreement of −0.48 to +0.50 years (total width <1 year). In contrast, both skeletal methods exhibited slightly wider but still acceptable agreement with chronological age (bias ≈ −0.07 to −0.08 years; limits ≈ ±1.5 years). These findings indicate that the Chandrasekar et al. [8] formulae are highly accurate and can be used interchangeably with the Fishman hand-wrist method for skeletal age estimation in North Indian children and adolescents, offering a reliable, radiation-efficient alternative (Table 5). The observed 95% limits of agreement between CVBA and the Fishman method (−0.48 to +0.50 years) fell well within the predefined ±1.0-year clinical acceptability threshold, supporting acceptable agreement.

**Table 5:** Bland-Altman analysis of agreement between the three age estimation methods A positive value indicates the first method overestimates compared with the second. SD: standard deviation, CVBA: cervical vertebral bone age

Comparison	Mean difference (bias) (years)	SD of differences (years)	95% limits of agreement (years)	95% limits of agreement width (years)
Chronological age − formula-derived CVBA	−0.08	0.74	−1.53 to +1.37	2.90
Chronological age − Fishman hand-wrist skeletal age	−0.07	0.76	−1.56 to +1.42	2.98
Formula-derived CVBA − Fishman hand-wrist skeletal age	+0.01	0.25	−0.48 to +0.50	0.98

## Discussion

This study evaluated the accuracy and applicability of the quantitative CVBA prediction formulae proposed by Chandrasekar et al. [8] in North Indian children and adolescents aged 4-18 years, using the widely accepted Fishman hand-wrist method as the reference standard [9]. The results demonstrated remarkable concordance between formula-derived CVBA, Fishman-derived skeletal age, and chronological age, with no statistically significant differences in the mean values and extremely high Pearson correlation coefficients. Bland-Altman analysis further confirmed the excellent agreement between the CVBA and Fishman skeletal age.

These findings are consistent with the original validation of the Chandrasekar et al. formula [8] in a South Indian cohort, where the authors reported correlation coefficients of 0.92-0.96 with chronological age and strong alignment with peak height velocity phases. The present study extends their applicability to individuals in North India, suggesting that the geometric ratios and regression equations derived from the C2-C4 morphology are robust across different regional subpopulations within India. Other authors have reported similar high accuracy of quantitative CVM methods. For instance, Caldas et al. [10] and Chen et al. [11] found that quantitative cervical vertebral parameters correlated strongly with hand-wrist maturity indicators in Brazilian and Chinese samples, respectively.

More recently, Byun et al. [12] validated a regression-based CVBA model in a Korean population using cone-beam computed tomography and found a statistically significant correlation with the hand-wrist method. The present results (mean difference 0.01 years, limits of agreement <1 year) are among the narrowest reported in the literature, reinforcing the superiority of multi-variable quantitative approaches over traditional qualitative CVM staging, which typically exhibits errors of 1-2 years and poor inter-observer reproducibility [13].

The absence of significant sex- or age-based subgroup differences is particularly noteworthy. Sexual dimorphism in vertebral maturation has been documented in some populations [14]. However, the sex-specific equations of Chandrasekar et al. [8] successfully neutralized this effect in our North Indian sample. Likewise, the formulae performed equally well across the pre-pubertal, pubertal, and post-pubertal cohorts, unlike earlier quantitative models that showed larger errors at the extremes of the growth curve. This uniform performance enhances clinical confidence when applying this method throughout the entire orthodontic treatment window.

Compared with traditional hand-wrist radiography, the CVBA approach offers a radiation-free alternative because C2-C4 are already visible on routine lateral cephalograms. Given that the "As Low As Reasonably Achievable" principle strongly discourages additional ionizing radiation in growing children, eliminating hand-wrist radiographs could reduce cumulative radiation exposure by 15-25% in patients requiring longitudinal growth assessment [15]. The excellent measurement reliability observed in this study further supports clinical adoption, as it minimizes the operator-dependent variability that has plagued subjective CVM methods. Although the present results suggest excellent agreement, they should be interpreted as indicative trends rather than definitive proof of universal interchangeability, given the study’s design constraints and population-specific sampling.

Clinical implications

The formula-derived CVBA can be confidently used in North Indian children and adolescents as a valid substitute for the Fishman hand-wrist method to determine skeletal maturity for timing orthodontic treatment (such as functional appliances, extractions, or rapid maxillary expansion), to modify growth in skeletal discrepancies, and to predict remaining growth potential. The implementation is straightforward using the existing cephalometric software. This approach is time-efficient, objective, and reproducible, and it eliminates additional radiation exposure, making it especially valuable in resource-constrained settings and for patients requiring serial assessments.

Limitations

This study has several inherent design limitations that must be acknowledged. The retrospective, single-center design and the absence of randomization and control groups limit causal inference. Although the sample size was statistically adequate, it remains modest and derived from a convenience-based orthodontic population, which may restrict external validity. Selection bias and residual measurement bias cannot be excluded entirely despite strict inclusion criteria and high examiner reliability. Blinding of examiners to chronological age during image assessment was not feasible due to the retrospective design. While no missing data were encountered, all statistical analyses assume normality and independence, which may not fully capture biological variability. These limitations warrant cautious interpretation of the findings.

## Conclusions

Within the limitations of this retrospective diagnostic study, the CVBA-derived quantitative formula may provide a reliable and radiation-efficient alternative to the Fishman hand-wrist method for assessing skeletal maturity in North Indian children and adolescents. The findings suggest a strong agreement between the two methods; however, broader application to other populations and clinical settings should be undertaken with caution. Further prospective, multi-center studies incorporating biological covariates such as BMI and growth patterns are recommended before routine universal clinical adoption.
